# Translating research into action: an international study of the role of research funders

**DOI:** 10.1186/s12961-018-0316-y

**Published:** 2018-05-24

**Authors:** Robert K. D. McLean, Ian D. Graham, Jacqueline M. Tetroe, Jimmy A. Volmink

**Affiliations:** 1 Faculty of Medicine and Health Sciences, Stellenbosch University, Tygerberg, South Africa; 2 Policy and Evaluation Division, International Development Research Centre, Ottawa, Canada; 3 School of Epidemiology and Public Health, University of Ottawa, Ottawa, Canada; 4 Centre for Practice Changing Research, Ottawa Hospital Research Institute, Ottawa, Canada; 5 Retired health researcher, Ottawa, Canada

**Keywords:** Knowledge translation, Knowledge mobilisation, Integrated knowledge translation, Research use, Research funding, Research evaluation, Research impact

## Abstract

**Background:**

It is widely accepted that research can lead to improved health outcomes. However, translating research into meaningful impacts in peoples’ lives requires actions that stretch well beyond those traditionally associated with knowledge creation. The research reported in this manuscript provides an international review of health research funders’ efforts to encourage this process of research uptake, application and scaling, often referred to as knowledge translation.

**Methods:**

We conducted web-site review, document review and key informant interviews to investigate knowledge translation at 26 research funding agencies. The sample comprises the regions of Australia, Europe and North America, and a diverse range of funder types, including biomedical, clinical, multi-health domain, philanthropic, public and private organisations. The data builds on a 2008 study by the authors with the same international sample, which permitted longitudinal trend analysis.

**Results:**

Knowledge translation is an objective of growing significance for funders across each region studied. However, there is no clear international consensus or standard on how funders might support knowledge translation. We found that approaches and mechanisms vary across region and funder type. Strategically tailored funding opportunities (grants) are the most prevalent modality of support. The most common funder-driven strategy for knowledge translation within these grants is the linking of researchers to research users. Funders could not to provide empirical evidence to support the majority of the knowledge translation activities they encourage or undertake.

**Conclusions:**

Knowledge translation at a research funder relies on context. Accordingly, we suggest that the diversity of approaches uncovered in our research is fitting. We argue that evaluation of funding agency efforts to promote and/or support knowledge translation should be prioritised and actioned. It is paradoxical that funders’ efforts to get evidence into practice are not themselves evidence based.

## Background

It is widely accepted that research can lead to improved health outcomes. However, uncovering health knowledge through research can be challenging, costly and time-consuming, and may fail to produce meaningful advances. Still, the expected positive benefits associated with knowledge creation have led governments, foundations and private institutions across the globe to dedicate envelopes of their tax-base or endowment to this endeavour. Around the world, recent estimates suggest that over US$ 100 billion is spent on biomedical research alone in a single year [1].

That being said, the benefits of knowledge are rarely achieved by its creation alone. Knowing what to do with health-related knowledge – how to access it, appraise it, tailor it for context, apply it in the practical world and know when it is not appropriate for practical application – is an entirely different challenge. Evidence indicates that health knowledge continues to be converted into practical applications in a slow and inconsistent way [2, 3]. The rush of publicly funded organisations to address this issue has been well documented in primary research and systematic reviews [4, 5]. Further, research funding agencies have also answered the call. Effectively, research funders have financed another charge for unlocking the power of knowledge, by increasing their focus on the conversion of knowledge into action. In this paper, we refer to this as knowledge translation (KT). Simply put, KT is the process of turning the knowledge that is generated in research studies into use in the real-world. For example, an improved product or device, a new policy or practice guideline, or a more accessible and thus equitable programme. The most broadly accepted definition of this process is offered by the Canadian Institutes of Health Research (CIHR), which uses the term KT to articulate to their Canadian constituency a support of:

“*A dynamic and iterative process that includes synthesis, dissemination, exchange, and ethically-sound application of knowledge to improve the health of Canadians, provide more effective health services and products, and strengthen the health care system.*” [2]

The primary objective of the research reported in this manuscript is to provide an international overview of the state of health research funders’ efforts to promote and support KT. To address this objective, the research takes a broad view, looking across an international sample of 26 funding agencies to identify trends and themes, rather than taking a deep dive into the particular practices of any single agency. In addition, the data collected and analysed in this manuscript builds on our previous work [6], which allows for longitudinal trend analysis. In our 2008 project, 33 health research funding agencies from around the world were reviewed to learn more about their KT roles and activities [6]. In this follow-up, we have replicated the study design from 2008, and have been able to include 26 of these agencies. A full account of our methods, findings and conclusions are provided in the following sections of this paper.

We believe the results of this study hold immediate practical value. The primary intended users of this study are health research funders. We hope this international stock-taking will facilitate evidence-informed reflection and debate amongst funders. Secondary users of this work will include health and science policy-makers, as well as researchers interested in KT (both those interested in the study of KT, but also those wanting to learn more about how they are being supported by funders to do KT).

### Why focus on funders?

Health research funders are just one of many actors in the pursuit of translating knowledge into action. Many actors, including but not limited to researchers, governments, patients, activists and taxpayers, have grown increasingly concerned with research being solution-oriented, and each plays an important role in ensuring an effective balance is struck between the creation of new knowledge and its application to health needs.

Research funders are rarely in the business of research implementation. However, we argue that the role of the research funder in KT is particularly pertinent. Funders control access to resources and therefore hold a position of power. This power can be used to stimulate and incentivise action on the part of the research community, including researchers, brokers and research users.

Evidence shows that funders contributing to KT have potential for impact. In spite of identified roadblocks in the knowledge-to-action pathway, studies examining the return on investment of public research have shown ample evidence of value [7, 8]. In simpler terms, research is a good investment in the public good. For example, one study performed by the Council of Economic Advisors to the President of the United States [9] demonstrated that the average social return on government investment in research and development was above 50%, a figure that was at the time, and still is, far greater than other areas of investment, including private sector research and development [9]. As impact assessment models that better capture the hard to measure ‘social impacts’ of research continue to evolve, we expect to see investments in research appearing even more attractive. Understanding how KT is supported by funders will help to ensure these investments generate the greatest return for people and society.

At the same time, the two primary stakeholders of the health research funder (the public and the academic community) have voiced clear interest in improved application and coordination of KT interventions by funders. The first stakeholder group, the public (often the research funder’s funder, and always the intended long-term beneficiary), has asserted a concern that governments pay greater attention to ensuring the practical utility of research they fund [10]. The second group, the academic community (the research funder’s primary beneficiary) has increasingly turned to funders to support their desire and build their capacity to meet this growing public call for knowledge translation [11, 12].

## Methods

### Study design

This research was undertaken with the intent of providing a comprehensive account of the international state of KT support offered by health research funding agencies in high-income countries. We adopted a longitudinal study design, wherein data from the same cohort was gathered across subsequent intervals in time. To do this, we purposefully grounded our study design in the work of Tetroe et al. [6] and McLean et al. [13].

#### Theoretical frameworks

Two theoretical frameworks were employed to support data analysis. One to categorise KT activities and a second to classify evaluation activities at funding agencies.

The first, in which we categorise, describe and provide our assessments of funding agency support for KT, is derived from the work of Lavis et al. [14]. In their 2006 paper, Lavis and colleagues propose a framework for assessing country-level efforts to link research to action with a view to “*inform country-level dialogues about the options for linking research to action*” [14]. This framework was applied in our data analysis as it provided an internationally recognisable system for KT activity classification and interpretation. The framework outlines three components into which we classify funding agency KT activities, these are (1) push activities, (2) pull activities, and (3) linkage and exchange activities (Box 1). For the reasons above, we believe this is a useful and rigorous means of analysing the KT activities of funding agencies. We caution that this framework does not facilitate a specific review of ‘priority-setting activities’ or ‘responsive grant-making’, i.e. when funding agencies strategically prioritise topics, disciplines or objectives for the research they fund. The KT activity classification framework employed here includes funding that has been ‘strategically prioritised’, but it does not examine the act of priority-setting separately.

To conduct an analysis of evaluation activities, we developed a new framework for funding agency evaluation activity classification. The framework is derived from theory and guidance on best practice in evaluation [15–19], as well as the work of Mintzberg on organisational strategy [20], and that of the Canada’s International Development Research Centre on how strategy and evaluation interact [21, 22]. Mintzberg argues that organisational strategy is not just what we say we do, it is also what we do [20, 22]. Because evaluation is used by organisations to understand what has been done, in essence, evaluation activities can be used to shine light on a picture of organisational strategy. In this research, we aimed to utilise this dynamic conceptualisation of organisational learning and strategic management by developing a framework for the analysis of funders’ KT actions.

Box 2 below provides further explanation of the framework that was developed and used for data analysis^1^. In short, it outlined three areas of strategy, namely (1) the ‘the intended strategy’ (what was planned), (2) ‘the realised strategy’ (what actually occurred), and (3) ‘the emergent strategy’ (the lessons learned and adopted into practice) of the funding organisation.

#### Longitudinal design and sampling frame

Our research was designed to provide a follow-up on the work of Tetroe et al. [6]. We drew our sample of funding agencies from the sample contacted in this 2008 study. With this approach we were able to undertake analysis of the same cohort at two discrete intervals in time. In the Tetroe et al. [6] study, a judgement sampling approach was employed to select funding agencies based on particular criteria of interest to the research team undertaking that study [23]. These criteria were (1) nationally scoped agencies and other disease-specific voluntary health organisations and (2) agencies that represented a continuum in or contrast in their KT support activities.

### Data collection protocol

#### Website reviews and agency templates

A data collection template was developed to gather information from the website and accessible publications of each funding agency. These templates were based on the data collected in the Tetroe et al. [6] study (keeping to our objective of conducting longitudinal analysis) and the theoretical categorisation of KT activities provided by Lavis et al. [14]. Templates were populated with information such as mandate, annual budget, types of KT support activities and KT evaluation activities. Following this initial web-based documenting process, the templates were sent via email to senior representatives of each agency for validation, updating and addition of data that were not available on the agency website or in accessible publications. The agencies were then asked to return the completed template to our study team, at which point a telephone interview was scheduled between the study research team and the agency.

#### Semi-structured qualitative interviews

We aimed to conduct telephone interviews with at least two representatives of each agency, including one senior representative of the KT function and one senior representative of the evaluation function. It should be noted that, in some cases, the two senior officials were the same individual, in some agencies an evaluation function did not exist, and in others a larger group of representatives desired to take part in the interview process and we agreed to this. The interview protocol was based on the completion of the agency template and a series of follow-up questions based on the flow of the discussion and emergent data of interest. This approach allowed us to complete the deductive learning exercise driven by the predefined template, but also to complement these data with new information on why and how any KT activities were being implemented in the eyes of the funding agency, and to allow the interviewer to probe further on issues of particular interest [24].

All interviews, except the CIHR interview, were conducted by telephone in either French or English. The CIHR interview was conducted in-person in the CIHR Ottawa, Canada, office. On average, interviews lasted between 30 and 90 minutes. Notes from any French language interview were translated into English by two bilingual members of the research team independently, and independent translations were cross-checked for consistency and accuracy. To minimise the threat of description or interpretation bias following the interview, the notes and the completed agency templates were returned to each agency for validation.

### Research ethics

Ethics approval for this project was granted by the Ottawa Hospital Research Institute, in Ottawa, Ontario, Canada.

## Results

The following section of the paper reports on the results of our research and preliminary interpretation. The section is divided into three parts. In the first, we present an overview of the 26 agencies included in the research. In the second, we explore the role of KT at the funding agency, in other words, how funding agencies have positioned KT for their agency in a qualitative and quantitative sense. In the third, we outline the KT initiatives being offered and undertaken at the funding agency, taking stock of and analysing actual KT strategies, programmes, funding mechanisms and evaluations of these efforts.

### Agency overview (region, focus, funding source, budget)

Table 1 provides a brief overview of the regions and agencies included in the study. It also displays those which were not in the study, but were involved in the t1 period research (from here forward the Tetroe et al. [6] project will be referred to as t1 and this project as t2). The seven agencies not included in this report were removed from the sample due to an inability to schedule an interview or an interview request being declined. Although it would have been possible to proceed with using publically available data, it was deemed that this would lead to questions about accuracy. Further, a brief description of the focus of each agency is included as well as the agencies’ overall annual budget at the time of contact.

**Table 1:** Overview of funding agencies studied

Country	Abbreviation	Organisation	Source of funds; Regional focus	Annual budget (CAD millions converted at time of contact)
Total Funding Agency Sample (*n* = 26)				
Australia (*n* = 3)	CCA	Cancer Council Australia	Charitable; National	16.5 (research specific)
	NHFA	National Heart Foundation of Australia	Charitable; National	71
	NHMRC	National Health and Medical Research Council	Public; National	774.5 (research specific)
Canada (*n* = 9)	AI (formerly AHFMR)	Alberta Innovates (formerly Alberta Heritage Foundation for Medical Research)	Public; Provincial	91.9
	CHSRF	Canadian Health Services Research Foundation	Public; National	15.2
	CIHR	Canadian Institutes of Health Research	Public; National	1000
	FRSQ	Fonds de recherche en santé du Quebec	Public; Provincial	100
	MSFHR	Michael Smith Foundation for Health Research	Public; Provincial	33
	CCRI (formerly NCIC)	Canadian Cancer Society Research Institute (formerly National Cancer Institute of Canada)	Charitable; National	41
	NSHRF	Nova Scotia Health Research Foundation	Public; Provincial	4.9
	SHRF	Saskatchewan Health Research Foundation	Public; Provincial	6
	SSHRC	Social Sciences and Humanities Research Council	Public; National	350.9
Netherlands (*n* = 1)	ZonMW	Netherlands Organization for Health Research and Development	Public; National	n/a
Denmark (*n* = 1)	FSS	Danish Agency for Science, Technology and Innovation – Danish Council for Independent Research – Medical Sciences	Public; National	44
Norway (*n* = 1)	RCN	Research Council of Norway	Public; National	1261
United Kingdom (*n* = 7)	AS	Alzheimer’s Society	Charitable; National	124
	CSO	Chief Scientist Office	Public; National	106
	HF	Health Foundation	Charitable; National	42
	NHS HTA	National Health Service – Health Technology Assessment	Public; National	14
	NIHR HS&DR	National Institute for Health Research; Health Services and Delivery Research	Public; National	18
	UK MRC	UK Medical Research Council	Public; National	1215
	WT	Wellcome Trust	Charitable; National	968
United States (*n* = 4)	AHRQ	Agency for Healthcare Research and Quality	Public; National	370 (research specific)
	NIH-NCI	National Institutes of Health – National Cancer Institute	Public; National	5300
	RWJF	Robert Wood Johnson Foundation	Charitable; National	400
	VA	U.S. Department of Veteran Affairs	Public; National	18
Agencies not included from Tetroe et al. [6] (*n* = 7)				
France	AFM	Association Française Contre les Myopathies	Charitable; National	n/a
	INSERM	Institute National de la Santé et de la Recherche Médicale	Public; National	n/a
	MOH	Ministry of Health: Programme Hospitalier de la Recherche Clinique	Public; National	n/a
Netherlands	ZN (Formerly CVZ)	Zorginstituut Nederland (formerly College voor Zorgverzekeringen)	Public; National	n/a
Sweden	SMRC	Swedish Medical Research Council	Public; National	n/a
United Kingdom	UK DH	United Kingdom Department of Health Policy Research	Public; National	n/a
United States	CDC	Centre for Disease Control	Public; National	n/a

### The position of KT at the funding agency

KT can be supported by a funding agency in a multitude of ways; however, the way that KT is positioned within the funding agency – explicit or implicit – is an important indicator of the level of significance it holds and the impact it may eventually have. We have used five measures to allow us to identify and investigate the intended role for KT at each of our sampled funding agencies. Then, by aggregating data, we can examine regional, global and longitudinal trends. Our five measures are (1) language used to describe KT, (2) mandating KT, (3) a senior agency members’ priority rating of KT, (4) human resources devoted to KT, and (5) financial resources devoted to KT. Results related to each of these measures are reported in turn in the following sections of the manuscript.

#### KT terminology

As a follow-up to the t1 study, we looked to identify terms that funding agencies were using to describe the concept of translating research into action – what we refer to in this paper as KT. Through agency website scans and follow-up interviews with agency staff, we identified a total of 38 terms in use by the 26 agencies studied.

In the t1 study, 29 terms were identified, and therefore, over time, there has been an increase in the number of terms used to describe the KT concept within funding agencies, even though the number of funders studied decreased by seven between the two study periods (Table 2). One might interpret this growth in terminology over time in a number of ways. On the one hand, it could be seen as a popularisation of the concept, as further definitions are being used. On the other, it could be interpreted as a lack of coordination and consistency in KT conceptualisation from one agency to another. We discuss our understanding vis-à-vis other findings of our research in the Discussion and Conclusions section of this paper.

**Table 2:** Terminology used to describe ‘Knowledge translation’ (KT) over time

Terms used to describe KT by participating funding agencies (as reported in interviews) in t1 and not in t2, *n* = 6	Terms used to describe KT by participating funding agencies (as reported in interviews) in t1 and t2, *n* = 23	New terms used to describe KT by participating funding agencies (as reported in interviews) in t2, *n* = 15
Applied health research Competing, coordination, co-optation Knowledge cycle Science communication The third mission Transmission	Capacity-building Diffusion Dissemination Exploitation Getting knowledge into practice Impact Implementation Knowledge communication Knowledge exchange Knowledge management Knowledge mobilisation Knowledge transfer Knowledge translation Linkage and exchange Popularisation of research Research into practice Research mediation Research transfer Research translation Teaching Translation Translational research Utilisation	Action cycle Application Coordination Dialogue Implementation science Knowledge movement Knowledge promotion Knowledge sharing Knowledge use Manualising interventions Policy and practice cycle Spread Scaling Translating research into practice Uptake

#### Mandating KT

**Fig. 1:**
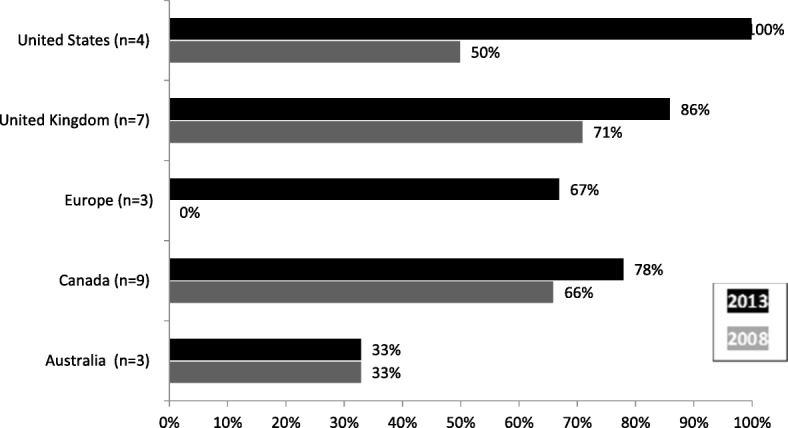
Change in knowledge translation inclusion in agency mandate over time

Perhaps the most palpable indicator of KT’s intended prominence at a funding agency is whether or not the agency includes the concept in its mandate. A mandate is the publically stated raison d’etre of an organisation, and is often legislated by an external body such as a Parliament or Board of Directors. To assess this, we scanned agency mandates to look for terms or concepts describing KT – not specifically the term knowledge translation. We validated our result for each agency in the follow-up interview with the funder. Of the 26 funding agencies involved, we identified that 20 (77%) included the concept of KT directly in their agency mandate. This result indicates that the majority of research funders in our sample publically declare that KT is a part of their core mission. However, data also indicates that the inclusion of KT in the mandate is an emerging trend. As Fig. 1 shows, the number of agencies including KT in their mandate has increased from t1 to t2.

Every global region studied demonstrates an increase in the inclusion of KT in funding agency mandates, save Australia, where mandate inclusion did not decrease but remained unchanged. At the individual agency level, we found that none of the agencies that included KT at t1 had removed the concept at t2.

Stemming from terminology and mandate reviews, we aimed to collect data on additional indicators of the intended role of KT at the health research funding agency. Table 3 displays the results for the three additional measures of KT positioning at funding agencies, namely (3) self-prioritisation of KT, (4) human resources devoted to KT, and (5) financial resources devoted to KT.

**Table 3:** Agency knowledge translation (KT) prioritisation, human resources and financial resources

Country	Agency^a^	KT prioritisation^b,c^	KT staff^c,d^	Annual budget for KT (CAD millions)^c,e^
Australia	CCA	n/a	1	2.9
	NHFA	‘High’	0	44
	NHMRC	5	80	n/a
Canada	AIHS	5	3	0.34 + embedded
	CHSRF	5	Embedded	Embedded
	CIHR	4	15	30 + embedded
	FRSQ	3 or 4	0	5
	MSFHR	5	2	0.45
	CCSRI	5	Embedded	Embedded
	NSHRF	5	1	n/a
	SHRF	4	0	Embedded
	SSHRC	n/a	2	24.5–31.6
Netherlands	ZonMW	n/a	20	n/a
Denmark	FSS	n/a	n/a	Embedded
Norway	RCN	n/a	n/a	Embedded
United Kingdom	AS	4	Embedded	Embedded
	CSO	n/a	1	0.62
	HF	3	Embedded	0.40
	NHS HTA	4	n/a	n/a
	NIHR HS&DR	3.5	2.5	2.4
	UK MRC	5	15–20	Embedded
	WT	5	45	Not fixed
United States	AHRQ	5	300	31
	NIH-NCI	4	7	n/a
	RWJF	5	35	340
	VA	5	Embedded	Embedded

^a^For the full agency names, please refer to Table 1.

^b^For KT prioritisation scores, a 5-point Likert scale was provided to the respondent. The scale was structured as: 5 – Very Important; 4 – Important; 3 – Neither important nor unimportant; 2 – Unimportant; 1 – Very unimportant

^c^No responses were forced in any part of this research, and so, in several instances ‘n/a’ is recorded as the data point ^d^‘Embedded’ was assigned to the ‘KT Staff’ column when the agency indicated KT is ‘a part’ of the duties of all, or a subset, of employees. Though none are assigned to it in particular

^e^The ‘Annual Budget for KT’ column includes funds reported by the agency for KT specifically. This may include funds for agency staff or KT activities such as grants or awards. Agencies themselves reported these figures, and we interpret that they are best positioned to have decided what counts as KT-specific funds for them; we caution that this does imply different uses of funds were being reported by different agencies

#### KT prioritisation

KT prioritisation is unique to this period of data collection. The intent of introducing this data point was to capture, and subsequently compare, a challenge which arose quite frequently in the qualitative aspects of the t1 research. The difficulty we observed was that agency representatives reported in the interview stage that KT was of a certain priority at their agency, although they did not have precise written policy, budgetary or other ‘hard’ documented evidence to support this claim. Given that our interviews were performed with senior officials of each agency (in many cases up to the VP level), and that the vision of a leader may potentially be used to judge the importance of an idea, we designed a simple categorical tool for the collection and classification of these claims.

In interviews, we discussed why and how they reached the agency score they did. We found that, when respondents were given the opportunity to explain the numeric rating given to their agency, they frequently asserted that KT was becoming an increasingly important global objective and that the interviewee’s individual funding agency was well-attuned to this trend and following suit. This finding is aligned to the data presented earlier demonstrating the growing trend of embedding the KT concept within the agency’s mandate. The generally high scores of KT prioritisation across agencies and regions indicate a trend of increasing KT importance within our cohort. However, when compared to other proxy measures of the ‘KT role’ at an agency (staff and budget), we did not see any particularly compelling patterns emerge.

#### Human resources devoted to KT

In examining human resources devoted to KT, each agency was asked to self-interpret and self-classify who was considered KT staff. Through semi-structured interviews, we then aimed to identify underlying reasons for these classifications. Interestingly, there is a divergence in who is defined as KT staff across agencies. To give an example, in the United States agencies, definitions of staff varied substantially. The Robert Wood Johnson Foundation held a broad view, including its nine evaluation staff as KT staff, suggesting that a focus on learning and programme improvement are both an evaluative duty and a part of the agency’s KT approach. In contrast, the National Institutes of Health National Cancer Institute reported its dedicated Implementation Science Team, a group that works directly on issues of KT conceptualisation and programming with the organisation and with its research community. Many agencies included communications groups in their calculation of KT staff. Further, the Agency for Healthcare Research and Quality suggested that the embedded nature of the KT work at their organisation meant that all employees should be counted as KT staff. This variation in interpretation of who constitutes KT staff was not restricted to the United States. We did not discern any trends in types of staff (e.g. communications, evaluation, considering all staff as KT) being classified as KT in one region but not in another.

Although not indicative of any generalisable difference in resource support (given our purposeful sampling approach and differences in regional sample characteristics), the data illustrates that the United States currently devotes the largest human and financial resource contributions to the KT objective at the funding agency level.

As human resources devoted to KT was not a variable collected in the t1 study, we were unable to perform any comparative analyses across time.

The over-arching finding of this line of analysis is that there is no generally accepted view of who constitutes KT staff at a research funding agency.

#### Financial resources devoted to KT

Given that financial resources devoted to KT were measured in both the t1 and t2 studies, we performed various comparative analyses on KT budget data received from each agency. However, none of these proved, in our view, to provide enough insight into KT spending trends at funding agencies to warrant further demonstration and/or data tables in this manuscript. Furthermore, we concluded that there was limited value in presenting changes in KT spending per region or per agency given multiple, significant confounding factors that would limit the ability to interpret such analysis (e.g. changes in total agency budgets versus KT budgets, currency inflation, regional variation in inflation, currency conversation and exchange rate fluctuations over time). That being said, one trend did emerge, namely that, across all regions, the number of agencies that could provide a precise budget figure for KT did not change significantly. In other words, the number of agencies earmarking KT funds remained generally the same across time.

For a closer look at this issue, we unpacked the more recent t2 data further. In summary, less than half of all agencies interviewed (11 of 26) were able to identify a specific amount devoted to KT. Ten of 26 reported that the KT spending of the organisation could not or should not be seen as an independent budget line, but instead that KT was embedded-in across the organisation’s expenditures. Seven of 26 agencies were unable to provide any funding details related to KT, which was in contrast with the fact that only one of 26 was unable to publically disclose any budget information. In sum, these data indicate that earmarking KT financial resources is not the norm across any region or the sample at large. To better understand the return on KT activities, this may be a useful area of data for agencies to track more closely in the future.

### KT initiatives

In this section, we turn to the specific programmes, mechanisms, modalities, activities, etc., that funding agencies were using to support KT.

**Fig. 2:**
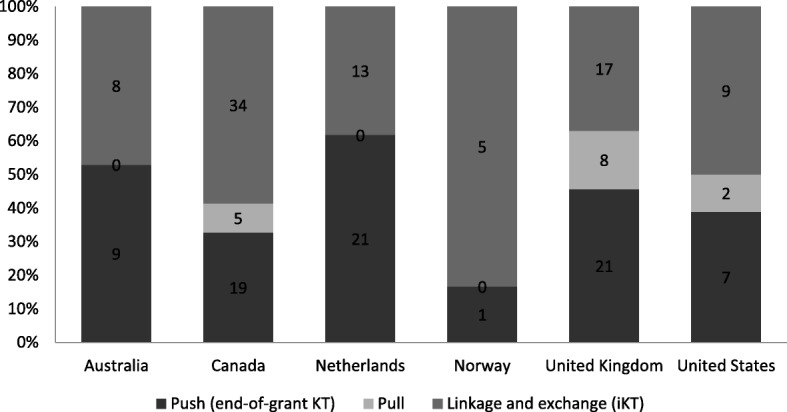
Number of push, pull, linkage and exchange programmes by country

Figure 2 presents the classification of agency initiatives across the three parts of our analytic framework, namely push, pull, and linkage and exchange (see Box 1 in the Methods section of this paper for a full description). Given the sampling approach employed, we caution against advanced quantitative comparative interpretation. We consider these data as categorical.

Most agencies favour linkage and exchange (or integrated KT (IKT)) and push efforts over pull efforts. There were a substantial number of IKT programmes supplementing the funders’ support for traditional programmes of curiosity-driven research. Qualitative interview data did not provide any clear conclusion on why these trends toward push and linkage and exchange efforts existed.

Although any regional analysis should be made with caution, a pattern does emerge with regards to programme distribution across category, namely that agencies and regions offer a general mix of programming, which is consistent with what is considered by Lavis et al. [14] to be a favourable approach.

**Fig. 3:**
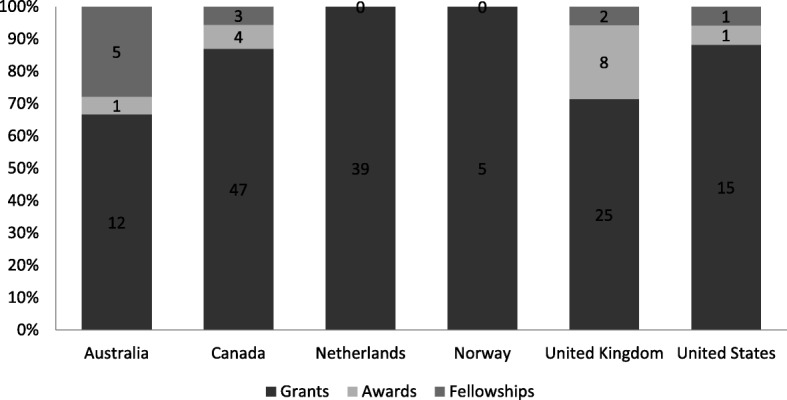
Number of knowledge translation (KT) grants, awards and fellowships by country

At t1, more attention was paid to the activities the funding agency required of researchers vis-a-vis the activities, either planned or unplanned, of the funding agency in support of KT. To address this, we restructured our classification of programmes in Fig. 3 by grants, awards and fellowships. Note that a single programme – the base unit in our above analysis (Fig. 2) – could include a series of grants or awards or fellowships. It should also be noted that Fig. 3 showcases programmes that were strategically designed for KT and does not include grants, awards or fellowships that were not designed specifically for KT, but may support KT due to the independent decision of a grantee to undertake KT.

The primary finding of this analysis is that the funding agencies are more involved in KT grant dispersion than other forms of KT support activity like awards or fellowships. To facilitate more precise interpretation, a comparison of how this trend in KT support varies from other areas within the health sciences would be a valuable area of additional study, e.g. investigating how the balance of grant, award and fellowship opportunities for KT compare to the balance of opportunities available for clinical trials, laboratory science, vaccinology and health systems research.

### Evaluation

The final area of findings reported in this manuscript describes an investigation of the evaluation of KT being conducted at funding agencies. Evaluation is selected as a focus area for two distinct reasons. First, KT evaluation has been identified as a significant gap in published expert opinion, theoretical research and empirical research [6, 14, 25–29]. Second, in the t1 research, there were no evaluations identified in any of the 33 agencies studied; however, nearly all 33 agencies articulated that plans and designs for evaluations were underway. As a result, a specific follow-up on progress with evaluation was prioritised for t2. In other words, an objective for t2 was to provide more than a stock-taking of programmes and practice at funding agencies, it was also to dig into the evidence guiding these efforts.

Given our specific focus for this study – KT activities/support at the funding agency – we purposefully reviewed evaluation undertaken at the funding agency level only, that is to say, an evaluation that focused on the KT programmes and activities of the funding agency. We did not include any evaluation being done by funded researchers in their own projects or the health interventions of others, even if this evaluation was funded by an agency in our sample (e.g. a large body of work being performed through the National Health Services – Service Delivery and Organization, e.g. [30, 31])^1^. Our aim was to learn about funding agency programmes and practice specifically. Table 4 illustrates evaluation activities being conducted of funding agencies’ KT programmes and practice; it utilises the Intended, Realised, Emergent (IRE) framework articulated in Box 1 of this manuscript.

**Table 4:** Intended → Realised → Emergent (IRE) Framework classification of agency knowledge translation (KT) evaluation activities^a^

	Intended strategy	Realised strategy			Emergent strategy
	Definition; KT objectives; KT implementation theory, etc.	KT evaluation/learning objectives	Evaluation methods for KT developed	Analysis and communication of findings	Uptake of evaluation evidence by funding agency^b,c^
Australia (3)	2/3	1/3	1/3	1/3	0/3
Canada (9)	8/9	5/9	3/9	3/9	1/9
Europe (3)	3/3	0/3	0/3	0/3	0/3
United Kingdom (7)	6/7	5/7	2/7	2/7	0/7
United States of America (4)	4/4	1/4	1/ 4	0/4	0/4
Total (26)	23	12	7	6	1

^a^This table includes only those activities focused specifically on KT and omits any which were considered ‘embedded’ in broader research quality assessments or operational reviews. This decision was based on agency representatives being categorically unable to elaborate what and how KT aspects and activities were ‘embedded’ in any broader evaluation when probed during interviews. This was corroborated in our review of the evaluation report or other related documentation. ^b^AIHS is the only agency to have completed a health research funding evaluation which they could demonstrate evidence to show it has been used to inform agency practice. ^c^After the completion of data collection, but before publication of this manuscript, CIHR completed and delivered its KT evaluation to its senior management committee; it is not included in the classification to uphold data consistency

Data indicates that funding agencies are putting considerable effort and resources into thinking through KT theories and objectives but much less into carrying out critical evaluations of these efforts/resources. Indeed, 23/26 funding agencies had a defined and planned KT strategy to some extent (recall 20/26 are currently including the concept in their agency mandate), yet only 7/26 had evaluated KT efforts and only 1/26 could demonstrate that evaluation results had been used to guide KT programmes or practice (i.e. to support evidence-based decision-making). In other words, a commitment to KT is evident, but learning-focused programming of KT was rare.

A deeper dive into the three components of the IRE framework helps to further understand the agencies’ KT strategy. See Box 2 in the Methods section of the manuscript for a full description of the IRE conceptual framework.

#### Intended strategy

In terms of ‘intended strategy’ there is a strong base of activity and effort in our sample of funding agencies. The majority of this effort was in setting a KT definition and outlining a series of KT goals. A minority of agencies had derived implementation theories (e.g. a theory of change) to describe the intended process and results of their KT efforts. One of these was the National Institutes of Health National Cancer Institute’s Implementation Science team. This agency has worked to articulate a theory of KT implementation, integrated a research translation continuum, and developed a set of contingencies and considerations into their KT support processes. Another example was CIHR, who had articulated a KT Funding Program logic model, and initiated an evaluation of the organisations strategic intentions for KT, by using this model in the evaluation design to outline expected KT results and critical assumptions.

#### Realised strategy

In terms of ‘realised strategy’, we have included all evaluation activities related to the assessment of realised organisational KT strategy. Table 4 indicates a decline in activities as we move from ‘intended’ to ‘realised’ strategy. Some insight into why this was the case was uncovered in the qualitative interviews. While the vast majority of agencies asserted that they deemed the evaluation of their KT funding to be a paramount endeavour, they also informed the research team that they did not have a firm grounding in how to undertake this task. Generally, it was suggested by agency representatives that research evaluation was a difficult undertaking; however, evaluating the translation of research into action was the most difficult component of this problem.

#### Emergent strategy

‘Emergent strategy’ is not surprisingly deficient when considered in relation to the trend of decreasing activity moving from ‘intended’ to ‘realised’ strategy documentation. At the time of data collection, only one agency (Alberta Innovates, a Canadian public Provincial funder) was using KT-specific evaluation results to inform decision-making and action.

## Discussion

As health policy, practice and programming continues to lag behind research-generated knowledge, KT remains a crucial objective within the health system. As has been argued in the past [32], the funding agency role in supporting KT has merit for a number of reasons. In this manuscript, we premise this argument on the position that incentive-setting power funders occupy, given the control they hold over financial resources. Playing the role of financier to the research enterprise places funders in an influential position to stimulate action around a particular topic such as KT. As Nobel Prize winning economist Joseph Stiglitz has recognised:

“*…the scientists whose research and ideas have transformed our lives in the past two hundred years have, for the most part, not been motivated by the pursuit of wealth. This is fortunate, for if they had, they would have become bankers and not scientists. It is the pursuit of truth, the pleasure of using their minds, the sense of achievement from discovery – and the recognition of their peers – that matters most. Of course, that doesn’t mean they will turn down money if it is given to them.*” [33]

### Positioning KT with funders

The purpose of this research was to take stock of how various funders are supporting KT and how well they are doing. In general, the 26 funding agencies whom we engaged demonstrated that KT is a high and a still growing priority. As mandates are changed (and maintained) to include the concept of KT, we interpret this to mean that governments and other health research funders are concerned with making research useful and actionable.

Previous studies on the role of the funding agency in KT (e.g. [6, 25, 34]) have argued that a common definition and/or classification of KT would be beneficial, and some suggest that a systematic framework to knowledge translation would contribute to conceptual clarity in the field [25]. We do not find evidence to disprove this hypothesis, but the findings of our research give us limited reason for concern. We suggest the diversity of experience across funders, by country, region, agency size and by agency type, is a representation of the diversity of context in which these organisations operate. We see no reason to conclude this is problematic. In our opinion, it is more than likely beneficial that programmes and strategies are contextually grounded.

At the same time, some trending in funder practice is evident. This study has identified further divergence in terminology over time (since 2008). It also uncovers a convergence of KT initiatives which funders are using to further their KT agendas. The prevalence of push efforts and linkage and exchange (or IKT) efforts, and the preference towards grants to support these, appears as a trend across our global sample. The emergence of IKT programming in particular represents a notable shift from traditional research funding approaches that have tended to favour the researcher over research users. Without robust evaluation data we cannot examine the evidence base for why these programmes and mechanisms are favoured, or appraise their effectiveness. However, we can offer some interpretation. First, and perhaps most likely, the similarity in programming and grant making activities might represent the emergence of an accepted framework for KT support based on informal exchanges between agencies. If this is the case, we note some concern that, in the absence of evaluations, the convergence of KT support activities may represent an emerging groupthink process rather than the co-development of a set of proven good practices. Secondly, although we would argue unlikely, these international patterns could be coincidental. Again, further evaluation would help to shed better light on the issue.

### KT evaluation – still, an area for action

In the 2008 t1 study, we identified a significant gap in funders’ execution and ability to execute evaluations of KT efforts. As a result, in this t2 research, we set an intentional focus on the issue of KT evaluation in order to learn how evaluation practice had evolved over time and to collect complete evaluations in order to investigate the possibility of synthesis and meta-evaluation.

We aimed to better understand and analyse evaluation activities by developing and applying the IRE framework (Box 2). This tool allowed us to identify, and subsequently investigate, evaluative and strategic strengths and weaknesses across agencies. In essence, the IRE framework enabled us to look beyond a simple count of evaluation reports or the potential undertaking of a synthesis of evaluation findings. Using the framework facilitated meaningful conclusions about the evaluation and strategy process. We would encourage those agencies who wish to improve their evaluation and strategy functions to consider a conceptual framework such as this, in particular for identifying areas for organisational improvement and/or cross-agency exchange, and we suggest the results of this research may provide some starting points. For example, applying the IRE framework highlighted how ‘intended KT strategy’ is relatively well developed across our sample of funders. Therefore, ‘intended strategy’ activities such as designing KT funding programmes, setting an organisational KT strategy, or developing KT theories of change, would be an immediately actionable and data-rich area of cross-agency learning and exchange.

Our data shows a substantial base of intended KT evaluation activities across the agencies and global regions in the sample. However, findings also highlight a significant lack of progress in undertaking targeted evaluations of KT, communicating results of these evaluations, and using findings of these evaluations to inform funder practice and policy. At the time of data collection, only one of the agencies in our sample had completed a targeted evaluation of KT. Though several agencies had evaluations that were underway or planned, it is important to recall the same was found in the t1 study (when nearly all agencies reported plans for KT evaluation). A clear conclusion is that evaluative data is not being used to measure progress against the objectives set out in earlier stages of KT programme design and planning. Given that the underlying objective of KT lies in moving evidence into action, it is paradoxical that the funders of KT do not employ this philosophy in their own work.

At the same time, this research has uncovered a lack of methodological know-how for evaluating KT as a major stumbling block for agencies who generally indicate a genuine interest in improving KT practice. As such, we suggest this is an area ripe for researcher (not just funder) focus. We also learned that funding agencies, themselves faced with budget austerity, do not always have the ability to make evaluations a priority, and especially the more challenging evaluations such as one focused on KT. Although we can understand this predicament, we do not agree that underfunding critical reflection is a sustainable cost-savings approach. We hope that identifying the persistent lack of KT-focused evaluation at funding agencies, both globally and across type of agency, will assist in kick-starting evaluative work. In our interviews with funding agencies, we heard great interest and genuine intention to undertake evaluations should the technical know-how be advanced and financial resources be available. We hope this research is used to support the cause.

### Study limitations

Although we are confident that our methodological approach allowed us to capture an accurate snapshot of KT activities at each agency, we caution that, because we did not interview all departments or branches of each agency, we cannot claim with absolute certainty that all KT activities have been identified.

The research reported in this manuscript has focused on the intentional efforts of funders to support KT. Focusing on the intentional may not have captured all KT activities supported. KT activities may go unreported when they have occurred as a part of a funding agency programme that is not intentionally supporting KT, particularly when they occur by decision of a grantee or awardee. For example, a researcher may decide to use a portion of a research grant to organise a meeting with hospital managers to discuss their findings; this activity has been technically supported by the funding agency with the grant, but it may not have been directed or recorded by the funding agency as KT support.

The longitudinal nature of the study design is weakened by the project team’s inability to include seven of the agencies included in the t1 study [6]. An inability for the research team to establish a contact at a particular funding agency meant we removed the agency from the sample. We did not want to rely on data collected from the web and document reviews alone. We have no reason to believe this has introduced any bias into the study, neither could we identify any characteristic or quality that removed agencies have eliminated or re-weighted in this sample vis-à-vis the 2008 sample.

A significant limitation stems from our sample of funding agencies being from high-income countries and focused on funding research that addresses high-income country needs. A review which includes low- and middle-income country funders was undertaken by Cordero et al. [25] as a companion to Tetroe et al. [6]. The follow-up to Cordero et al. [25] will be critical to understand the global story of funding agency support for KT^2^.

A limitation in our study design relates to where we have focused attention in data collection and analysis. We have purposefully taken a broad view with this research, engaging 26 funding agencies from around the world in the study, allowing the identification of broad trends and themes for KT practice at research funding agencies. However, it does not facilitate the deep investigation of a particular funding agency’s experience with KT.

We note, for reader interpretation, the timing between data collection and publication – data were collected from funding agencies for this project in 2012/13 (9 to 10 years after the data collection in t1). Readers’ should interpret the findings accordingly.

## Conclusions

In summary, our research confirms that KT is an objective of growing significance for the health research funders across the high-income regions of Europe, Australia and North America. The findings demonstrate that there is no clear-cut standard or practice for implementing KT at a funding agency. We suggest that KT is an idiosyncratic matter that relies on the many contextual factors presented to a particular research funder. There is very likely no viable one-size-fits-all solution. We suggest that the diversity of experience this research has uncovered indicates that any sweeping conclusions or directives for KT at funding agencies should be handled with caution, and also calls for evaluation of KT in these different funder contexts to learn what works, for what type of funder and why.

We suggest that the critical evaluation of KT should be prioritised and actioned so that evidence-based decision-making becomes not only the objective of KT programmes, but also a part of how these programmes operate and evolve. These evaluations should take into consideration the particular context of the agency that undertakes the evaluation, and should make this context clear in order to facilitate other agencies’ interpretation of the results. To kickstart and advance high-quality evaluation, we suggest funders support KT evaluation experimentation, innovation and collaboration among each other on the topic. Funders should not feel alone, this effort may well embrace the researcher community interested in doing and improving KT. As the significance of KT grows for funders, so must the evidence base to guide it.

## Box 1 Knowledge translation activity classification framework

**Push** – activities and programmes targeted at the ‘pushing’ of research-produced knowledge into the hands of appropriate knowledge users – users who may not have otherwise been aware of the research and its implications. Examples include research communications, funding opportunities or funder activities, or typical end-of-grant funds that an agency may provide to a researcher to encourage the dissemination of findings such a publication in an Open Access journal, or the creation of a plain language findings brief.

**Pull** – activities and programmes that facilitate knowledge users’ access to research results. For example, a forum where researchers are brought to discuss an issue of importance with identified knowledge users.

**Linkage and exchange** – activities and programmes that support the establishment of partnerships between researchers and knowledge users through multiple parts of the process of research design, execution and/or dissemination. Linkage and exchange is also referred to as integrated knowledge translation and co-creation/co-production [35–37]. An example would be a research grant that required both a researcher and a knowledge user to apply in partnership for funding, representing a break with the traditional researcher curiosity-driven approach to science. This more participatory approach may also involve non-researchers (e.g. patients, clinicians, managers, etc.) as reviewers in the peer-review process.

Note: This is a very brief description of these well-developed concepts of promoting research use. Further reading on the subject could include Lomas [38] and Lavis et al. [39].

## Box 2 Intended → Realised → Emergent (IRE) framework for strategy classification

**Intended strategy** (the planned knowledge translation (KT) strategy) – Includes actions such as defining KT, setting clear KT objectives or goals, mapping these objectives within internal and external structures, mechanisms and constraints (in a realist or classical empiricist way), defining stakeholders (intended and unintended), drawing identified factors into implementation theories for KT programmes.

*Evaluator’s use ‘intended strategy’ to first understand programme purpose and to then construct measures for programme or organisational assessment. Robust evaluations will consider intended strategy during methodological design. Typically, these intentions are developed by evaluators in constructs such as ‘theories of change’, ‘logic models’, ‘logframes’, etc. All of these tools hold the same general purpose of articulating a programme’s intended strategy and results in more depth and detail than a stated objective.*

**Realised strategy** (the KT strategy that was executed) – Includes the actual KT programmes, initiatives and activities of the funding agency.

*Evaluators use these elements as the ‘object of assessment’ or ‘evaluand’. Evaluation activities related to this will include actions such as designing evaluation studies, monitoring and collecting data, analysis and interpretation of data and communicating findings. Here, evaluations would identify KT support that was working as intended and that which was not as well as unpacking the mechanisms, contexts and systems that govern success.*

**Emergent strategy** (KT strategy evolving from evaluation use) – Includes the broad range of actions related to using evaluation findings in KT programme refinement, development, overhaul or cessation (i.e. evidence-based decision-making). In other words, the evidence-based direction that an agency embarks upon.

*Evaluators produce knowledge related to a realised strategy that, through the complex process of uptake and implementation, is built into the re-thinking of strategy (e.g. confirming status quo, course correction or complete cessation). In the evaluation literature, this is referred to as ‘evaluation use’ and is manifested in evidence-based action.*

## Abbreviations

CAD: Canadian dollar
CIHR: Canadian Institutes of Health Research
IKT: Integrated Knowledge Translation
IRE: Intended, Realised, Emergent
KT: Knowledge Translation

## Footnotes

1 There were many evaluations conducted by the research community through the CLARHC programme of the National Health Services – Service Delivery and Organization, we reference two examples. None of these evaluations focused on the activity of the funding agency in support of KT.

2 This study is currently in design by authors: RKDM, JAV, IDG.
